# Supplementary material for: The probability of edge existence due to node degree: a baseline for network-based predictions
Source: Gigascience. 2024 Feb 7;13:giae001. doi: 10.1093/gigascience/giae001 (PMC10848215; doi:10.1093/gigascience/giae001)
Supplement: giae001_Response_to_Reviewer_Comments_Original_Submission [file giae001_response_to_reviewer_comments_original_submission.pdf]

Author's Response To Reviewer Comments

We revised this in a document with formatting. This system appears to remove the formatting, so I've attempted to delineate comments with a "Comment:" prefix and responses with a "Response:" prefix.

Reviewer #1:

Comment: The manuscript "The probability of edge existence due to node degree: a baseline for network-based predictions" presents novel work. But some of the sections are written very briefly, so it is difficult to understand. The section that needs revision are: Degree-grouping, The edge prior encapsulates degree, Degree can underly a large fraction of performance and Analytical approximation of the edge prior. The result section needs revision.

Response: We updated our phrasing in each section with the goal of making our phrasing more clear. Our changes expand key areas of these sections for improved readability.

Comment: Some other concerns are:
Academic adhar, Jaccard coefficient, preferential atachment etc are link prediction methods. Why auther has termed them as edge prediction features.

Response: It is our understanding that the terms edge and link are interchangeable. For example,
A graph in this context is made up of vertices (also called nodes or points) which are connected by edges (also called links or lines). [Quoted from Wikipedia]
The terms "arc," "branch," "line," "link," and "1-simplex" are sometimes used instead of edge (e.g., Skiena 1990, p. 80; Harary 1994). [Quoted from Wolfram Mathworld]
We use the term edge consistently throughout the paper.

Reviewer #2:

Comment:I n this manuscript, the authors introduce a network permutation framework to quantify the effects of node degree on edge prediction. The importance of degree in the edge detection task is self-evident, and the quantification of this effect is undoubtedly groundbreaking. The experimental results on a variety of datasets demonstrate the advanced nature of the method proposed by the authors. However, some parts require further explanation from the authors and can be considered for acceptance in a later stage.
1. The imbalance of the degree distribution has a significant impact on the results of the edge detection task. In this manuscript, the author proposes a framework to quantify this impact. It is important to note that the manuscript does not explicitly mention the specific form in which the quantification is reflected, such as whether it is presented as an indicator or in another form. Therefore, further explanation from the author is needed to clarify this aspect.

Response: We thank the reviewer for this suggestion. We did not sufficiently highlight the advantage of our approach. In short, the approach is to compare any desired network or edge prediction metric to the distribution of this metric across random, permuted graphs. This can be done at any level and using any metric, as permuted graphs are identical in everything but content to the original. We have modified our phrasing in the paper to make this more clear.

Comment: 2. The authors propose that researchers employ marginal priors as a reference point to discern the contributions attributed to node degree from those arising from specific performance. It would be helpful if the authors could elaborate further on the methodology or provide a sample demonstration to clarify the implementation of this approach.

Response: We updated the phrasing in our introduction. There was some ambiguity between the edge prior and the network permutation approach for other edge prediction features. Edge priors quantify edge

probability given degree alone. Permuted networks can also be evaluated using edge prediction methods to generate a per-node-pair distribution of an edge prediction feature, against which the feature's true value (in the unpermuted network) can be compared.

Comment: 3. For the XSwap algorithm, I wonder that if the authors could provide a more detailed explanation of its workings, including a step-by-step implementation of the improved XSwap. Furthermore, it would be beneficial if the authors could highlight the significance of the improved XSwap algorithm in biomedical tasks.

Response: We provided a diagram of XSwap in our revision. The final point is discussed further below in our response to point 7.

Comment: 4. The author presents the pseudocode of the XSwap algorithm in Figure 2, along with the improved pseudocode after the author's enhancements. Both pseudocodes are accompanied by explanatory text. However, I believe that expressing them in the form of a figure would make it more visually appealing and intuitive.

Response: We agree the reviewer's suggestion is an excellent one. We included a brief diagram of XSwap in our revision.

Comment: 5. The authors introduce the edge prior to quantify the probability of two nodes being connected solely based on their degree. I request the authors to provide a detailed explanation of the specific implementation of the edge prior.

Response: We updated our phrasing to read as follows:
The edge prior can be estimated using the fraction of permuted networks in which a given edge exists. In short, for a given node pair (a, b), given N permutations of the network, and given that m of these permutation contain (a, b) , the prior for (a, b) is m / N, which is also the maximum likelihood estimate for the binomial distribution success probability.

Comment: 6. In the "Prediction tasks" section, the author utilizes three prediction tasks to assess the performance of the edge prior. It is recommended to segment correctly for better display of the content.

Response: We thank the reviewer for pointing out this oversight. We have split paragraphs in that section according to the prediction task.

Comment: 7. The focus of the article might not be prominent enough. It is advisable for the author to provide further elaboration on the advanced nature of the proposed framework and its significance in practical tasks. This would help emphasize the main contributions of the research and its relevance in real-world applications.

Response: We appreciate this comment and agree that our focus was not expressed clearly enough. We trimmed and rephrased the abstract and the manuscript itself. We hope that these changes have made our purpose more clear.
