# Supplementary material for: The probability of edge existence due to node degree: a baseline for network-based predictions
Source: Gigascience. 2024 Feb 7;13:giae001. doi: 10.1093/gigascience/giae001 (PMC10848215; doi:10.1093/gigascience/giae001)
Supplement: giae001_Reviewer_2_Report_Original_Submission — Linlin Zhuo -- 6/24/2023 Reviewed [file giae001_reviewer_2_report_original_submission.pdf]

Reviewer Report

Title: The probability of edge existence due to node degree: a baseline for network-based predictions

Version: Original Submission **Date:** 6/24/2023

Reviewer name: Linlin Zhuo

Reviewer Comments to Author:

In this manuscript, the authors introduce a network permutation framework to quantify the effects of node degree on edge prediction. The importance of degree in the edge detection task is self-evident, and the quantification of this effect is undoubtedly groundbreaking. The experimental results on a variety of datasets demonstrate the advanced nature of the method proposed by the authors. However, some parts require further explanation from the authors and can be considered for acceptance in a later stage.1.The imbalance of the degree distribution has a significant impact on the results of the edge detection task. In this manuscript, the author proposes a framework to quantify this impact. It is important to note that the manuscript does not explicitly mention the specific form in which the quantification is reflected, such as whether it is presented as an indicator or in another form. Therefore, further explanation from the author is needed to clarify this aspect.2.The authors propose that researchers employ marginal priors as a reference point to discern the contributions attributed to node degree from those arising from specific performance. It would be helpful if the authors could elaborate further on the methodology or provide a sample demonstration to clarify the implementation of this approach.3.For the XSwap algorithm, I wonder that if the authors could provide a more detailed explanation of its workings, including a step-by-step implementation of the improved XSwap. Furthermore, it would be beneficial if the authors could highlight the significance of the improved XSwap algorithm in biomedical tasks.4.The author presents the pseudocode of the XSwap algorithm in Figure 2, along with the improved pseudocode after the author's enhancements. Both pseudocodes are accompanied by explanatory text. However, I believe that expressing them in the form of a figure would make it more visually appealing and intuitive.5.The authors introduce the edge prior to quantify the probability of two nodes being connected solely based on their degree. I request the authors to provide a detailed explanation of the specific implementation of the edge prior.6.In the "Prediction tasks" section, the author utilizes three prediction tasks to assess the performance of the edge prior. It is recommended to segment correctly for better display of the content.7.The focus of the article might not be prominent enough. It is advisable for the author to provide further elaboration on the advanced nature of the proposed framework and its significance in practical tasks. This would help emphasize the main contributions of the research and its relevance in real-world applications.

Level of Interest

Please indicate how interesting you found the manuscript: Choose an item.

Quality of Written English

Please indicate the quality of language in the manuscript: Choose an item.

Declaration of Competing Interests

Please complete a declaration of competing interests, considering the following questions:

- Have you in the past five years received reimbursements, fees, funding, or salary from an organisation that may in any way gain or lose financially from the publication of this manuscript, either now or in the future?
- Do you hold any stocks or shares in an organisation that may in any way gain or lose financially from the publication of this manuscript, either now or in the future?
- Do you hold or are you currently applying for any patents relating to the content of the manuscript?
- Have you received reimbursements, fees, funding, or salary from an organization that holds or has applied for patents relating to the content of the manuscript?
- Do you have any other financial competing interests?
- Do you have any non-financial competing interests in relation to this paper?

If you can answer no to all of the above, write 'I declare that I have no competing interests' below. If your reply is yes to any, please give details below.

I declare that I have no competing interests

I agree to the open peer review policy of the journal. I understand that my name will be included on my report to the authors and, if the manuscript is accepted for publication, my named report including any attachments I upload will be posted on the website along with the authors' responses. I agree for my report to be made available under an Open Access Creative Commons CC-BY license (http://creativecommons.org/licenses/by/4.0/). I understand that any comments which I do not wish to be included in my named report can be included as confidential comments to the editors, which will not be published.

Choose an item.

To further support our reviewers, we have joined with Publons, where you can gain additional credit to further highlight your hard work (see: https://publons.com/journal/530/gigascience). On publication of this paper, your review will be automatically added to Publons, you can then choose whether or not to claim your Publons credit. I understand this statement.

Yes Choose an item.
